# Model-Driven Processing of Passive Seismic While Drilling Data Acquired Using Distributed Acoustic Sensing Without Conventional Drill-Bit Pilot Measurements

**DOI:** 10.3390/s26030768

**Published:** 2026-01-23

**Authors:** Emad Al-Hemyari, Roman Pevzner, Konstantin Tertyshnikov

**Affiliations:** 1 Centre for Exploration Geophysics, Curtin University, GPO Box U1987, Perth, WA 6845, Australia; konstantin.tertyshnikov@curtin.edu.au; 2 Mineral Exploration Cooperative Research Centre (MinEx CRC), Drilling Analytics Research Centre (DARC), Curtin University, 3 De Laeter Way, Bentley, WA 6102, Australia

**Keywords:** DAS, passive seismic, seismic while drilling, SWD processing

## Abstract

This article presents an advanced processing workflow for a Seismic While Drilling (SWD) dataset acquired using Distributed Acoustic Sensing (DAS) in a cross-well setting at the Otway International Test Centre (OITC) in Victoria, Australia, where no pilot signals were recorded. Recording the drill bit signature enables and simplifies the decoding of passive seismic signals emitted by the drill bit while drilling. In conventional SWD, a measured drill bit signature is used to correlate passive seismic recordings and to determine source trigger times, analogous to vibroseis processing. Without this reference, both source timing and signature must be inferred from the recorded wavefield. This can typically be achieved by backpropagating the recorded seismic data over short time windows, estimating the source location and trigger time based on the peak RMS energy in space and time. However, to simplify the processing of SWD data, a data processing workflow is presented, guided by travel time and seismic modelling, which transforms passive SWD data into active equivalents. The transformed data can then be used to characterize the subsurface by implementing travel time tomography and cross-well imaging. The results demonstrate reliable velocity and structural information can be recovered from DAS-based SWD data without pilot measurements, enabling simplified and scalable deployment of passive seismic while-drilling workflows.

## 1. Introduction

Surface seismic data acquisition requires the mobilization of substantial equipment, which negatively impacts productivity and thus increases overhead costs and associated risks. Eliminating active vibroseis sources would eliminate their related costs and operational human risks. In a drilling environment, using the drill bit as an alternative seismic source is a passive seismic technique known as Seismic-While-Drilling (SWD) [1,2,3]. This technique, also known as the drill bit SWD, has been used steadily since the 1980s to infer subsurface geological information. The drill bit SWD is not to be confused with VSP while drilling (VSP-WD), introduced by Schlumberger in the early 2000s, which occurs while drilling, using seismic receivers within the drilling bottom hole assembly (BHA) and an active source on the surface [4].

Several SWD methods have been developed to deduce subsurface geological information for various geophysical applications, including the mining industry [5]. SWD has been investigated extensively by geophysicists in the seismic industry. Focus on source characterization dominated the early developments preceding the introduction of the passive seismic technique. In the case of SWD, this was achieved through measurements and analysis of drill bit vibrations [6,7] and modelling of its energy emissions [6,7,8]. These developments paved the way for using the drill bit as a seismic source [9] and developing the reverse VSP concept [10,11]. Such developments predominantly focused on the roller-cone drill bit from the late 1960s to the mid-1990s before the introduction of Polycrystalline Diamond Compact (PDC) bits in the early 1990s. The drilling community has mainly motivated them and continues to play a significant role in multiple areas, such as real-time drilling optimization, monitoring and improving drilling equipment wear and drill-string dynamics [12,13,14]. Further investigations of the drill bit as a seismic source followed, specifically to understand its energy radiation patterns [15]. More case studies successfully applied reverse VSP (RVSP), walk-away RVSP [16,17] and cross-well setting [18]. By then, some of the main challenges related to the drill bit energy imbalance and the non-repeatability of drilling conditions had emerged [19,20]. Despite these limitations, these developments have led to improved utilization of SWD data for imaging around the well [21,22].

In the early 1990s, researchers had already gathered enough experience defining requirements for successful SWD recordings where sufficient drill bit energy is observable. Besides the type of drill bit being used, the energy emitted is related to the formations being drilled and the drilling parameters being used [3]. Another challenge of SWD is that the drill bit signature or pilot is unknown, given that it is a passive source. The development of downhole measurement sensors while drilling enhanced the understanding of seismic emissions from drill bits and later matured into tools to record the drill bit pilot signature [3]. Their initial utilization did not require high data rate recording and telemetry, as they were intended for optimizing drilling operations and improving BHA designs. This limitation, in turn, restricted their use to memory-based recordings retrieved post-drilling. Although they have been developed to withstand harsh downhole conditions, they still lack precise downhole clocks, further complicating their use [23,24]. Nonetheless, they still contributed to understanding drill bit emissions and alternative pilot signal measurements at the surface, on or near the drilling rig [3,25,26].

These methods commonly deploy receivers on the surface or downhole within nearby wells to record the energy emitted during drilling. Recording the drill bit signature or pilot is crucial for decoding recorded drill bit signals and determining the source emission time [9,27]. To imitate active seismic surveys, most existing methods require a dedicated sensor along the drill string to record the pilot signal for cross-correlation with other receivers. However, cross-correlation may pass undesired noise from the pilot signal to the correlated records [28]. Additionally, adding sensors on the drilling rig or within the drilling string is often an engineering challenge and is prone to quality issues in the presence of surface drilling activities [29].

The use of top-drive pilot sensors in earlier applications paved the way for wired-pipe telemetry [27,30]. Thus, this allows for the successful real-time recording and retrieval of the downhole pilot [31]. Typically, wired-pipe telemetry comes at a much higher cost, thus reducing its use, especially in onshore drilling, where economics are much tighter. Where neither rig nor downhole pilot recordings are available, good-quality drill bit signals could still be retrieved using a geophone measurement close to the drilling rig [32,33]. The pilot measurements are typically used to correlate with geophone data. Pilot signal autocorrelations in depth are used for time corrections of the pilot-geophone correlations to produce RVSP gathers. So far, all the methods discussed above have used source pilot measurements or approximations to imitate controlled sources at known emission times. Without source pilot measurements, well logs and prior knowledge of seismic velocities in the area can be used for calibration [34]. In the latter case, data-driven methods are one way to estimate the source signature from passive measurements. Another approach is to use seismic interferometry with inter-source or inter-receiver variations [35,36]. Time-reversal methods are also used for passive seismic identification and characterizing subsurface scatterers [37,38,39]. These methods try to estimate the unknown emission time of the source.

Ultimately, the goal of any seismic exploration is to provide structural images and properties of the subsurface at an acceptable resolution to delineate potential petroleum or mineral deposits. For the case of mineral deposits, their scales and depths are different depending on their types. For example, gold deposit thicknesses can vary immensely, from thin veins to meters within quartz reefs or sulfide stringers, often concentrated at bedrock interfaces within alluvial systems (a few meters thick) or within broad paleochannels (tens of meters thick) [40]. Similarly, iron ore deposit thicknesses also vary significantly by type, from thin layers (few meters thick) to massive Banded Iron Formations and Channel Iron Deposits (tens of meters thick) [41].

## 2. Data Acquisition

At the OITC in Victoria, Australia, multiple drilled wells, instrumented with fiber-optic cables, were previously drilled at the site to monitor CO2 injection, making them ideal for continuous monitoring. In 2019, during the preparation for Stage 3, a deviated well, CRC-4, was drilled using a downhole motor and a PDC bit up to a vertical depth of 1650 m [42].

### 2.1. Passive Data Acquisition

The CRC-4 well was drilled from the same drill pad as the previously drilled CRC-3 vertical well, which was instrumented with an engineered fiber-optic (FO) cable, containing a Silixa Constellation fiber (Silixa, Elstree, UK), and installed behind the casing. Similarly, another vertical well, CRC-2, was previously drilled and instrumented with a single-mode fiber-optic cable. The CRC-4 well was drilled in two runs: a shallow section and a deep section. In a cross-well configuration, as detailed in Table 1, a continuous DAS dataset was recorded within the CRC-2 well while drilling the shallow section of the well. Another DAS dataset was recorded within CRC-3 well while drilling the deeper section of the well, which will be the focus of this article. A Silixa iDAS v3 interrogator (Silixa, Elstree, UK) was used to acquire the DAS data within the two recording wells, first while connected to the CRC-2 well, then to the CRC-3 well. Continuous recording yielded passive seismic records of 30 s in length, sampled at a rate of 1 millisecond. No recording of the drill bit pilot signal was performed, which complicates processing the acquired passive data.

### 2.2. Acquisition Geometry

**Figure 1:**
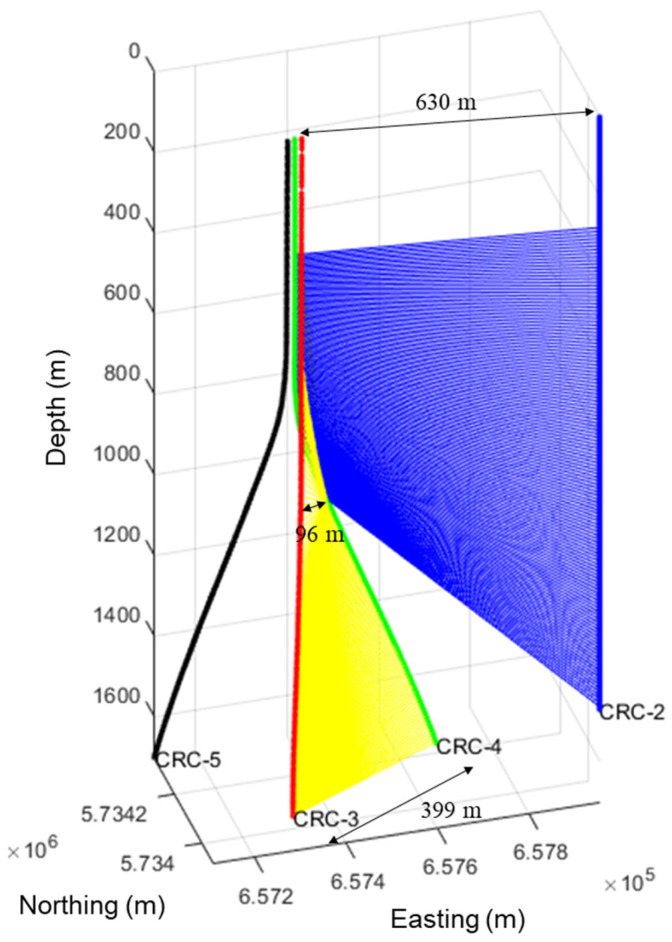
Wells diagram showing the SWD datasets acquisition geometry within the CRC-2 well while drilling the shallow CRC-4 well section, and within the CRC-3 well while drilling the CRC-4 well deep section. All subsequently drilled wells were mostly deviated (CRC-5 shown for demonstration). The yellow and blue areas highlight the ray-paths between the well being drilled, CRC-4, and the receiver wells, CRC-2 and CRC-3, used for recording while drilling the shallow and deep sections, consecutively.

Figure 1 shows well diagrams of the three wells used to acquire the SWD datasets. The drilled well, CRC-4, is plotted in green, while the two monitoring wells, CRC-2 and CRC-3, are plotted in blue and red, respectively. The CRC-2 well, used to record the CRC-4 shallow section DAS data, is around 630 m from the top of the CRC-4 well. The top of the deeper section of CRC-4 is offset by around 96 m, and the bottom by 399 m from the CRC-3 well. While engineered fibers had gratings every five meters, the recording was performed every 1 m, using a gauge length of 10 m, which resulted in massive DAS datasets.

### 2.3. Drilling Data

**Figure 2:**
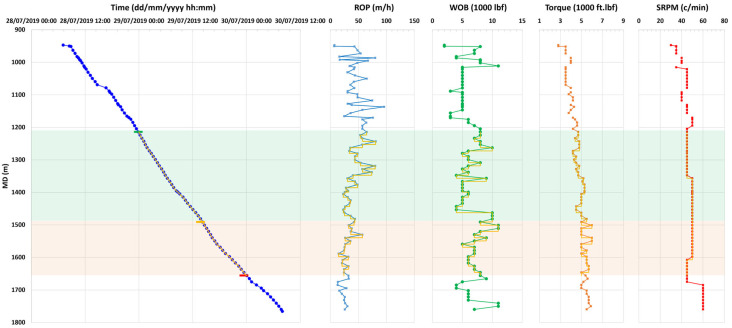
The drilling rig data acquired during the drilling of the CRC-4 well, against measured depth (MD), and including the drilling date and time in blue, rate of penetration (ROP) in light blue, weight on bit (WOB) in green, torque on bit in orange, and drill bit rotations per minute (RPM) in red. The yellow curves represent the upscaled drilling data for each 30 s seismic record. The green and orange colors areas highlight good quality and degrading quality data against measured depth.

The rig sensors collected several useful drilling data such as the measured depth (MD), weight on bit (WOB), torque, and drill-bit rotation rate (RPM), which are used to calculate the rate of penetration roughly every 5 min, as shown in Figure 2. The drilling rig data is not sampled regularly, and does not change within 30 s, which is the length of the seismic records. Hence, they have been resampled to have values for each seismic record. The resampling is performed in measured depth, then translated into corresponding times. The drilling rig data helped quality-check subsequent data processing steps and assisted in guiding the data processing. The rig data was correlated against amplitude attributes calculated from the recorded DAS data.

## 3. Seismic Modelling

**Figure 3:**
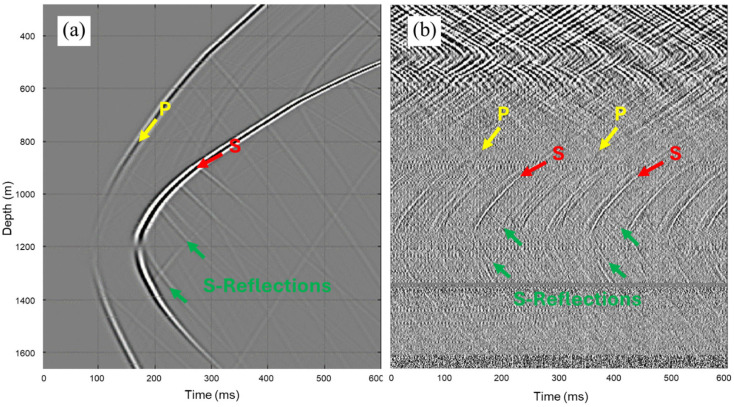
A comparison of (**a**) modelled and (**b**) recorded seismograms at a source depth of 1200 m.

A sample record, from a drill-bit elevation of around 1200 m, in Figure 3b, shows the repetitive nature of the SWD data. Different wave modes are generated as the drill bit interacts with the rock formations being drilled. PDC bits work by shearing rocks as they rotate under the application of weight. As a result, more shear waves (S-waves) are observed in the recorded data than primary waves (P-waves). To investigate the possibility of locating the source in time and space without needing a recorded reference pilot signal, and to guide the processing of the SWD data, forward modelling is performed mimicking the recorded data acquisition geometry.

### 3.1. Model Building

**Figure 4:**
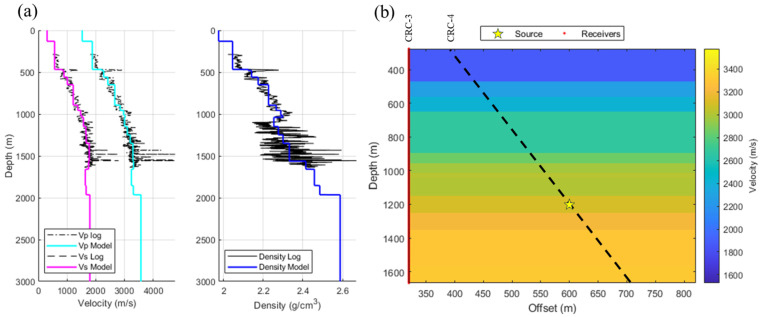
Model building for travel time and seismic modelling, where (**a**) shows upscaled 1D elastic property models from a CRC-3 well log, and (**b**) the extended 1D P-wave velocity model to 2D with an overlay of the CRC-3 well equipped with DAS receivers and the drilled CRC-4 deviated trajectory.

To facilitate a better understanding of the wavefield generated by the drill bit, a representative 1D layered velocity model was created using well logs and formation boundaries from the monitoring CRC-3 well. This model was then used to generate an upscaled 1D model of P-wave and S-wave velocities and density, as shown in Figure 4a. Figure 4b shows the acquisition geometry of the receivers, spanning a depth range of 277–1667 m, in the CRC-3 well, with a source at a depth of 1200 m, overlaid on a P-wave velocity model extended to 2D.

### 3.2. Travel Time Computations

**Figure 5:**
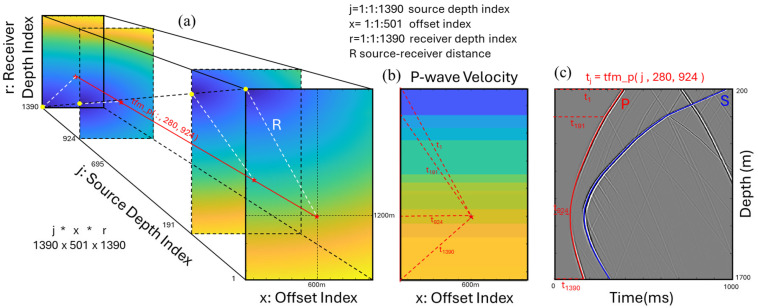
Travel-time computation using the fast-marching method generating (**a**) a 3D volume for each source depth, offset, and receiver depth indices, (**b**) a P-wave velocity profile with an overlay of a source at depth of 1200 m and 600 m offset, and (**c**) a modelled seismic record for the same source with an overlay of the computed P-wave and S-wave direct arrival times.

The generated P-wave and S-wave 2D velocity models are used as initial models to calculate travel times from all possible source locations within these models to the vertical receivers. An implementation of the fast marching method was used to calculate the travel times, which approximates the solution to the Eikonal equation [43]. This implementation generates 3D volumes of P-wave and S-wave travel times for all receiver depths, source depths and offsets from the monitoring well, as shown in Figure 5. For validation, the computed travel times are overlaid on a simulated seismogram for a source at a depth of 1200 m and 600 m offset from the monitoring well. The computed travel times are used as time corrections in addition to guiding the processing of SWD data.

### 3.3. Elastic Modelling

Elastic modelling is performed for a source located at a depth of 1200 m from the Kelly Bushing along the well trajectory, denoted by a yellow star in Figure 4b, with a Ricker wavelet of 50 Hz peak frequency. For receivers in the vertical monitoring well, a 1 m spacing is used over depths ranging from 277 to 1667 m, as denoted by the red dots in Figure 4b. To simulate a non-uniform source radiation pattern, a single drill bit source excitation is simulated by combining vertical and horizontal sources at the same subsurface location. A weighted sum of the receiver vertical components of vertically and horizontally oriented sources is computed and then differentiated with respect to depth to simulate the strain recording by DAS. Figure 3a,b show a side-by-side comparison of the resulting modelled data and an actual recorded record of a drill-bit source at the same depth. The two seismograms appear similar, apart from their different onset travel times and the repetitive nature of the SWD data. Yellow and red arrows indicate P-wave and S-wave direct arrivals, respectively, while green arrows indicate S-wave reflections from formations above the source depth. The seismogram in Figure 3b shows additional wavefield components related to surface waves travelling within the borehole and recorded by shallow receivers, which are very difficult to eliminate by processing. Other types of noise are observed, such as zero-amplitude DAS channels, random noise and channel striping, which are typical of DAS data. Luckily, these types of noise are easier to correct for or eliminate.

The modelling exercise not only helps in demonstrating the kinematics of the wave propagation but also serves as a demonstration of the source mechanism because of the interactions of different wavefields, collocated vertical and horizontal sources, which is clearly apparent in the polarity reversal of the S-wave direct arrivals, as shown in Figure 3a. It is also important to note that once all simulated particle velocity seismograms are converted into strain by derivation along the fiber length, their direct arrivals polarities change, which is purely due to the nature of the receivers, and not the source radiation pattern.

## 4. Drill Bit Source Characterization

Using a passive source presents challenges, especially since it is uncontrollable. The effective use of passively recorded data requires the locations of the passive sources and their trigger times, which are unknown and must be estimated from the data.

### 4.1. Reverse-Time Method

Time reversal methods backpropagate the recorded wavefield to its point of origin in space and time. For the simplest case of acoustic wave propagation in inhomogeneous media, time reversal can be explained as follows [44]. The wave equation for the pressure field p(x,t) at the receiver location x is

(1) $$\nabla \left(\frac{\nabla p}{\rho }\right)=\frac{1}{\rho v^{2}}\frac{\partial^{2}p}{\partial t^{2}}$$

where v(x,t) and ρ(x,t) are the velocity and density, respectively. If p(x,t) is a solution to this wave equation, $p\left(x,-t\right)$ is also a solution; however, it is a non-causal one. Therefore, the solution is limited to $p\left(x,T-t\right)$ where *T* is the total recording time, this solution requires recording the wavefield at each subsurface location x over 0≤t≤T interval before retransmitting it in reverse time, which is non-feasible. Thus, measuring the wavefield and its normal derivative at a limited portion of the medium allows for calculating it at all other points within the medium using the Kirchhoff integral, which becomes more realistic. The approach follows Huygens’s principle, which states that every source with a propagating wavefront generates secondary sources at all points within the medium. Backpropagation with the actual medium velocity focuses the wavefield at the primary source location and t corresponding to the direct wave arrivals. The wavefield would be out of focus at positive and negative onset times. The application of the time-reversal method is illustrated using elastic modelling of seismic data.

### 4.2. Source Location Estimation

**Figure 6:**
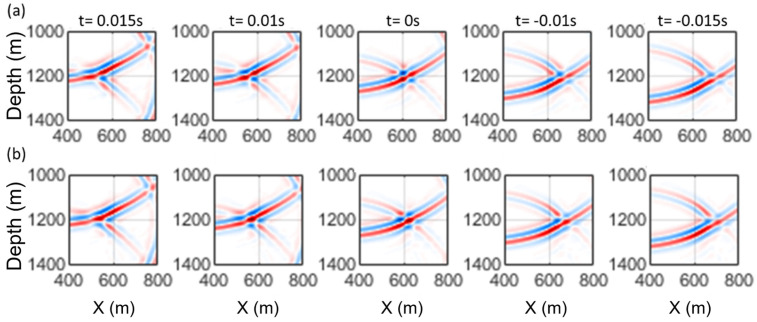
Wavefield focusing comparison around the source location between the (**a**) before and (**b**) after source radiation pattern correction. Each snapshot amplitudes have been normalized for better comparisons, with red and blue corresponding to positive and negative values.

The modelled synthetic seismogram in Figure 3a was backpropagated using the actual P-wave velocity model. Figure 6a demonstrates the time-reversal energy focusing for the modelled wavefield, where time is advancing from left to right. The wavefield energy is concentrated at t = 0 s, where the source is located, and is defocused elsewhere. As the modelled data show, the primary arrivals have opposite polarities above and below the source depth, resulting in destructive interference at the exact onset time and source location. A simple radiation pattern correction is demonstrated in the following subsection.

**Figure 7:**
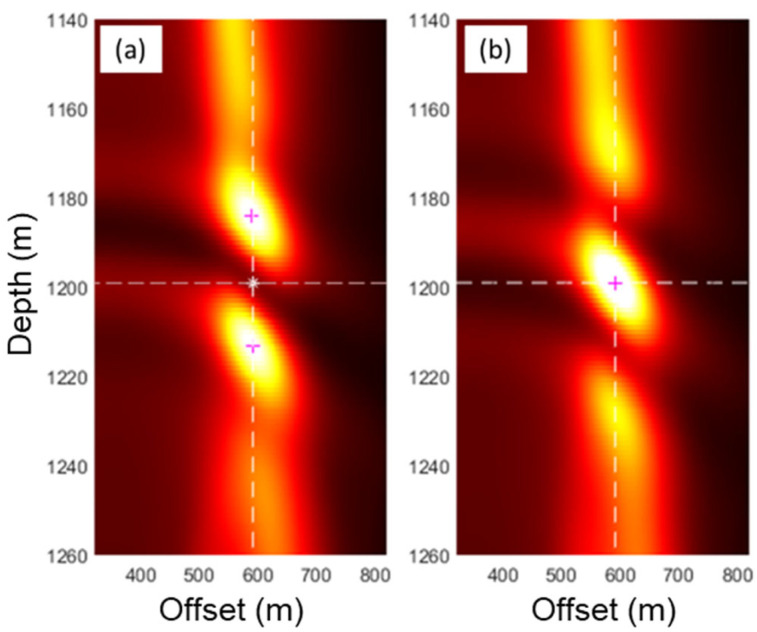
Accumulated RMS energy over 100 ms of propagation time (**a**) before and (**b**) after source radiation pattern correction.

This source radiation pattern did not impede the source location estimation, as the energy focusing is better indicated by calculating the maximum RMS energy over an extended propagation time. Figure 7a shows an image of the RMS energy summed over a 100-millisecond propagation time around the actual onset time. The white asterisk indicates the midpoint between the two maxima of the energy sum indicated by magenta crosses. This midpoint estimates the source location in space and depth, which in this case occurs at a depth of 1199 m and an offset position of 591 m from the monitoring well. The estimated source location is within 9 m of the actual source location, located at a depth of 1200 m and an offset of 600 m.

Similar results were obtained using the S-wave velocity, which primarily contributed to a more accurate source location estimation in the offset direction.

### 4.3. Source Radiation Pattern Correction

Alternatively, a simple radiation pattern correction is implemented by reversing the polarity of arrivals below the source during backpropagation, resulting in constructive interference, as shown in Figure 6b.

To better compare the two results, the maximum RMS energy is calculated over an extended propagation time around the onset time for each case. Figure 7 shows two images of the RMS energy summed over a 100-millisecond propagation time around the actual onset time. In Figure 7a, the white asterisk indicates the midpoint between the two maxima of the energy sum indicated by magenta crosses. In contrast, Figure 7b shows a single maximum due to the implemented radiation fix. In both cases, the midpoint on Figure 7a and the single maximum on Figure 7b estimate the source location in space and depth, which in this case occurs at a depth of 1199 m and a horizontal offset of 591 m. The estimated source location is within 9 m of the actual source location, located at a depth of 1200 m and an offset of 600 m. Similar results were obtained using the S-wave velocity but are not shared here.

## 5. SWD Data Processing

**Figure 8:**
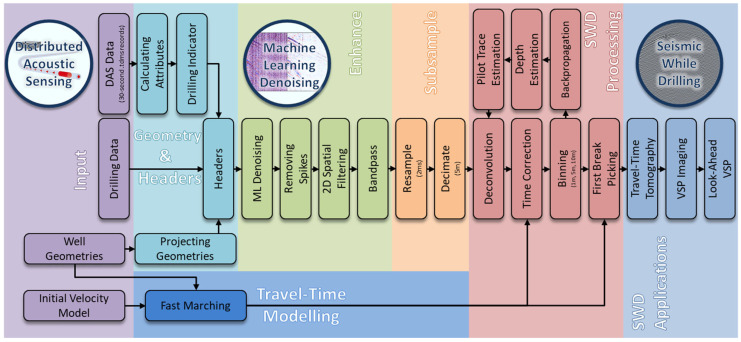
A specialized Seismic While Drilling data processing workflow, integrating different datasets, leveraging advanced machine learning denoising, and travel time modelling.

A specialized seismic while drilling data processing workflow was developed, as shown in Figure 8, starting with data integration and encompassing both traditional and machine learning denoising. As continuous DAS recordings generate massive datasets, adequate decimation is performed in both space and time for efficient processing. Subsequently, a model-based approach was developed to transform the passive SWD data records into active equivalents in preparation for seismic while drilling applications, such as cross-well travel time tomography and imaging. The developed approach does not require recording the drill bit signature; instead, it estimates it from passive data. The approach also employs an iterative method to enhance data transformation, thereby increasing confidence.

### 5.1. Data Preparation and Integration

First, all the input data are prepared, including the DAS data, rig drilling data, and source-receiver geometries. An initial velocity model from the CRC-3 well is also prepared to perform the seismic and travel time modelling.

#### 5.1.1. Amplitude Attributes

**Figure 9:**
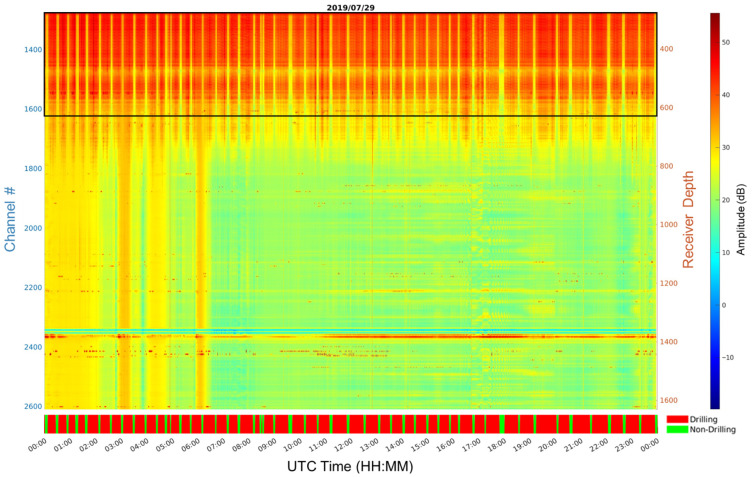
An RMS amplitude attribute map over a day of recording with an overlay black window over the shallow DAS channels used to compute a drilling indicator shown at the bottom.

Amplitude attributes are calculated for each DAS receiver channel and all records, as shown in Figure 9. These are then used to develop a drilling indicator to help narrow the processing to records where drilling occurred. The drilling indicator was calculated using the mean RMS Amplitude of shallow channels, as indicated by the black rectangle in Figure 9. The drilling indicator red color indicates drilling, while the green color indicates no drilling.

#### 5.1.2. Acquisition Geometry Projection

**Figure 10:**
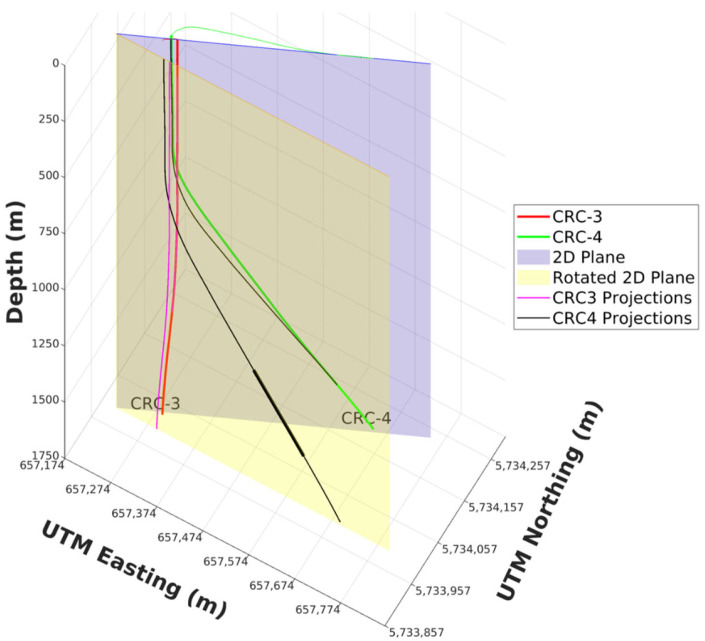
The original source-receiver geometries are projected into a 2D plane (purple) and rotated into a plane (yellow) parallel to the easting direction to simplify subsequent processing steps.

The receiver array geometry was determined from the deviation survey of the CRC-3 well and the DAS recording channel offset. Similarly, for the sources, geometries were determined from a combination of the deviation survey of the CRC-4 well and the measured depth from the rig drilling data. To simplify the data processing and tomography steps later, the original geometries of the sources and receivers were projected into a 2D plane, which was then rotated to be parallel to the Easting coordinate, as shown in Figure 10.

#### 5.1.3. Geometry and Headers Assignment

These projected geometries and the original geometries are included in SEG-Y headers, along with the drilling indicator and drilling data. The receivers’ geometry was determined from the deviation survey of the CRC-3 well and the DAS recording channel offset. Similarly, for the sources, geometries were determined from a combination of the deviation trajectory of the CRC-4 well and the measured depth from the drilling rig data.

### 5.2. Data Enhancement and Denoising

The passive seismic data goes through a step of denoising and enhancement to improve subsequent processing outcomes. As previously shown in Figure 3b, passive DAS data are contaminated with strong-amplitude surface waves travelling within the borehole and recorded mainly by shallow receivers, which are very difficult to eliminate by processing. This noise is handled by omitting channels shallower than 800 m in depth. Other types of noise, such as zero amplitude DAS channels, random noise, and channel striping, which are typical of DAS data, are easier to correct or eliminate. These types of noise will be the focus of this subsection.

#### 5.2.1. Machine Learning Denoising

**Figure 11:**
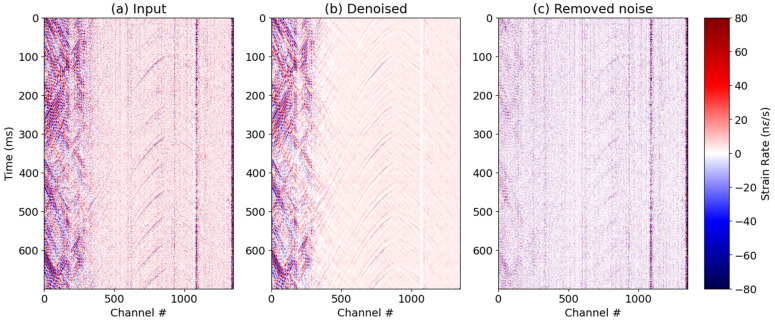
A comparison of (**a**) raw DAS and (**b**) machine learning denoised records, and (**c**) the removed DAS noise.

First, a machine learning denoising algorithm that eliminates most of the optical DAS noise was applied to all the DAS data [45]. This approach was based on supervised Learning, where synthetic data with added real DAS noise, recorded in an acoustically isolated setup, was used to train a U-net network with three layers. The training of the network aims to establish a mapping between the noisy and clean synthetic data, enabling the removal of noise at the application step. This step addresses the DAS optical noise, which appears as random noise and vertical striping due to variable fiber-optic coupling. Figure 11a shows a raw passive gather from a drill bit source depth of around 1200 m, while Figure 11b shows the same passive gather after the machine learning denoising, and Figure 11c shows the removed DAS noise. The machine learning denoising yields the most improvement to the data with minimal signal leakage. Subsequently, it is followed by multiple denoising steps targeting different types of noise, such as spikes, horizontal striping, and unusable low frequencies.

#### 5.2.2. Traditional Denoising and Filtering

Remaining amplitude spikes are localized in time and frequency, making them easy to remove without affecting the remaining record. A frequency-domain denoising algorithm is applied to the data, balancing spectrograms based on their median values and a calculated threshold value over a 50-trace window. A 2D spatial filter is then used to remove noise in the data, which appears as horizontal striping across all channels at specific recording times. This filter performs alpha-trimmed mean filtering over a 51-trace window and the whole length of each trace. The final denoising step involves applying a bandpass filter to remove very low frequencies in the continuously recorded data, thereby stabilizing subsequent processing steps.

### 5.3. Data Decimation

Even though the engineered fiber is made with gratings ideal for recording every 5 m, the data acquisition measured every 1 m, resulting in a huge dataset. The acquired data is decimated from a 1 m receiver sampling to 5 m by vertically stacking every five traces, while ensuring no aliasing, which also serves as an additional step in denoising random noise. The records are then resampled from 1 millisecond to 2 milliseconds to conserve storage space and accelerate computations. This step reduces the data size by a factor of ten.

### 5.4. Specialized SWD Processing

The subsequent processing step, which is the most critical, aims to transform every continuous and repetitive record at each drill bit depth into something equivalent to active seismic with a source located at the drill bit depth, where the seismic energy is focused into single P-wave and S-wave arrivals and their reflections. In an ideal scenario, where a synchronized pilot signal is recorded, it is used to correlate with and then deconvolve each seismic record, mimicking the process used with active-source (vibroseis) data processing. The key enabler here lies in synchronizing the pilot signal recording, which enables adequate time corrections for the correlated records and references them to the appropriate zero time. As a pilot signal was not recorded here, it is estimated from the data.

#### 5.4.1. Pilot Signal Estimation

One clear observation from this dataset is that it consistently exhibits stronger, coherent signals on receivers above the drill bit source [46,47]. A 2D correlation sliding window was used to generate a correlogram, allowing for the selection of an appropriate data-driven pilot trace depth estimation. In this case, the best pilot trace depth was around 170 m shallower than the source depth, throughout all records. An estimated pilot trace is derived for each 30 s record.

#### 5.4.2. Deconvolution

The estimated pilot traces were used for deterministic deconvolution to focus the repetitive data around direct arrivals. As the PDC bit predominantly generates shear waves, the zero lag is centered around the highest energy, which occurs around the S-wave direct arrivals, requiring time correction.

#### 5.4.3. Time Correction

Once deconvolution is done, time-delay corrections are applied. These time corrections are determined using travel time modelling with an initial velocity model and known geometries. The time correction for each record is computed based on expected S-wave travel times, for that source depth and relative to the pilot trace depth. Each of the deconvolved records is then time-shifted by its calculated time shift. At this stage, the passive data records have already been converted to their active equivalents, incorporating the correct travel times. These transformed records can then be backpropagated to get refined source depth estimates. Additionally, these transformed records are now better suited for the application of a deep learning implementation to estimate the source location and subsurface properties [48].

**Figure 12:**
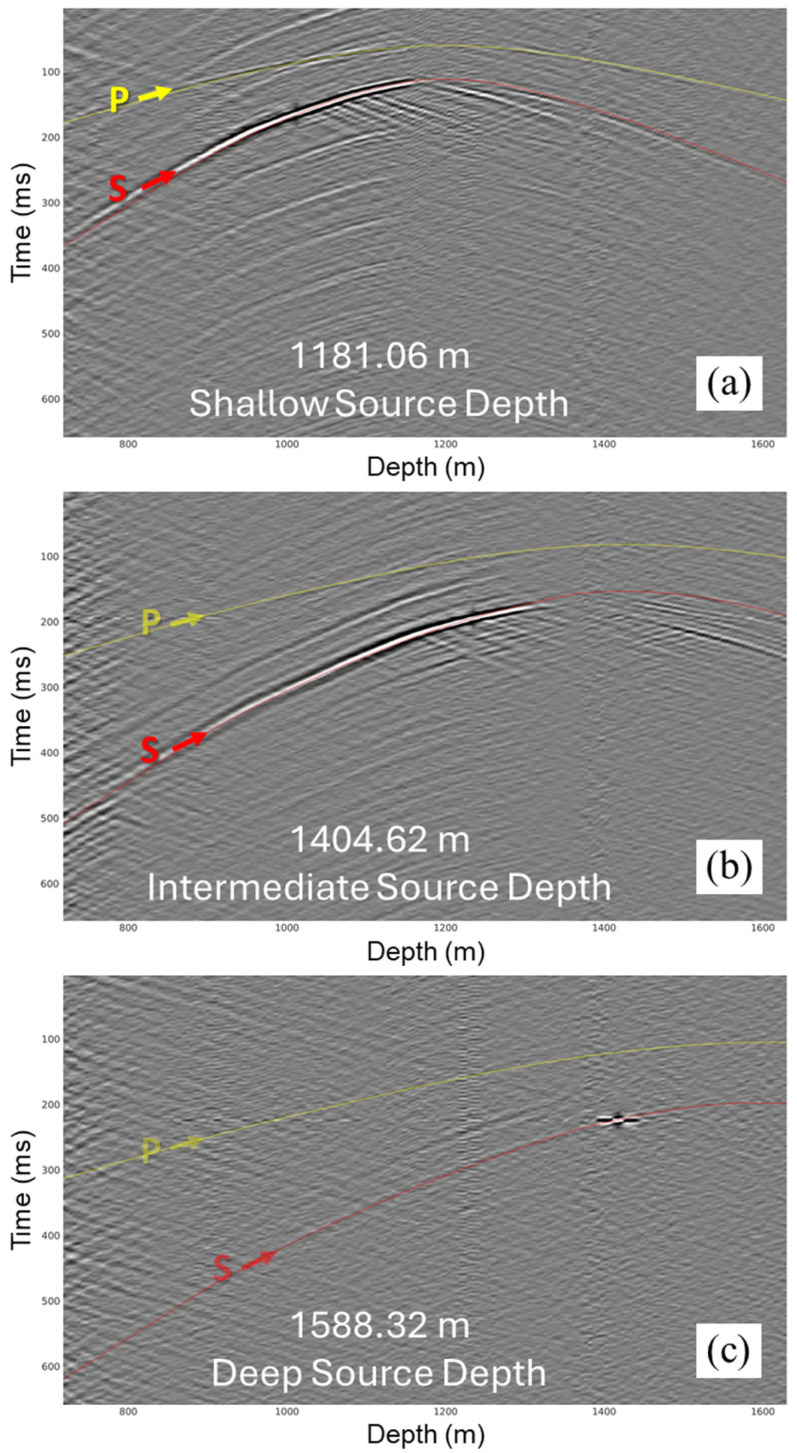
Transformed passive records into active equivalents for (**a**) shallow, (**b**) intermediate, and (**c**) deep source depths with overlays of P-wave and S-wave direct arrival travel times.

Figure 12 shows three deconvolved records at three different source depths (shallow, intermediate, and deep). It is observed that as the source depth increases, direct arrivals begin to diminish and eventually disappear beyond a certain depth. This is mainly due to deviating geometry of the drilled well with respect to the monitoring well and the lower rate of penetration. Thus, generating lower acoustic energy that cannot be detected easily.

#### 5.4.4. Source Depth Binning

**Figure 13:**
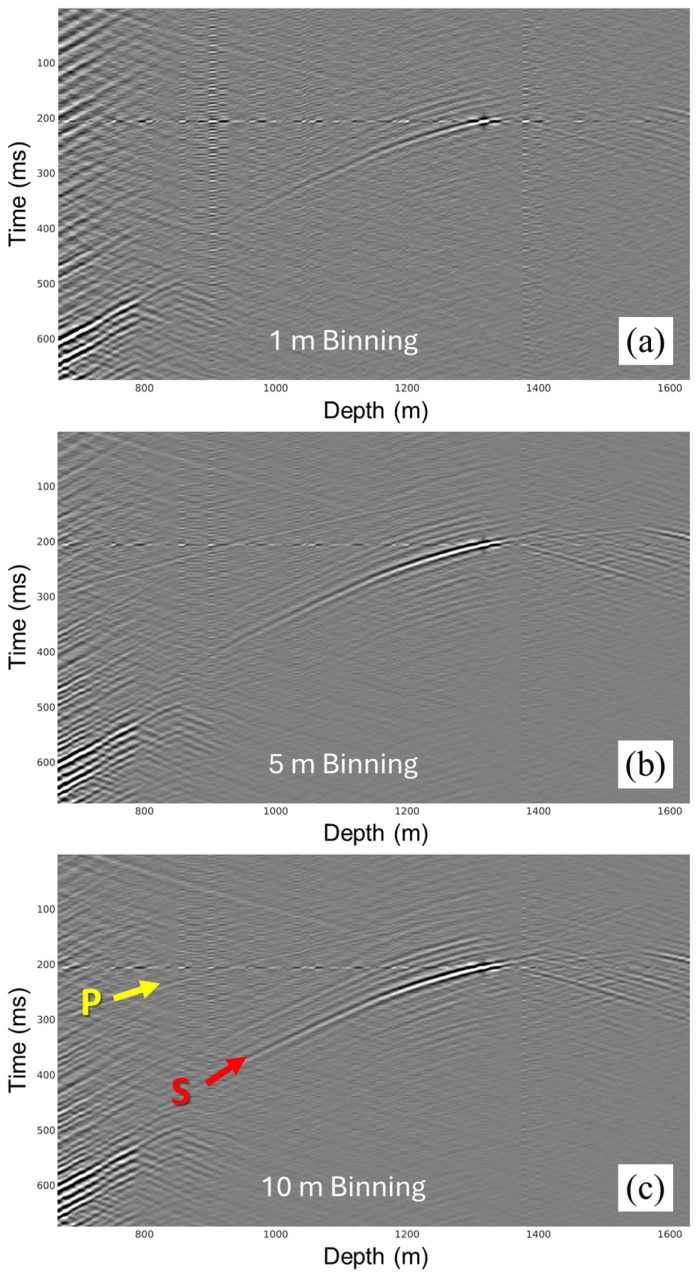
Binned records at a deep source location of 1546 m using (**a**) 1 m, (**b**) 5 m, and (**c**) 10 m depth bin sizes.

To correct for the dimming of direct arrivals with increasing source depth, multiple records are stacked over different measured source depths along the drilled well trajectory to enhance the signals. Different depth bins of 1, 5, and 10 m are tested. The number of stacked records for different bin sizes at each depth would be higher with larger bin sizes. A good correlation between the 1 m bin fold and the calculated ROP serves as a reliable processing quality check step. A record from a deep source depth, which typically has the poorest data quality, is used to illustrate the effect of binning on the data. Subsequent processing steps, such as first-break picking and imaging, require good-quality data. Figure 13 shows seismograms of binned source gathers, at depth of 1546 m, at three different bin sizes (1, 5, and 10 m), where the direct arrivals of P-waves and S-waves become more prominent when stacking more records within a larger bin size of 10 m. Now that binned gathers are of better quality, an optional step to run them through backpropagation to obtain refined depth estimates and fine-tuned velocities, to update pilot signal estimates, rerun deconvolution and apply new time delay corrections. Ultimately, the iterative framework ensures minimizing the propagation of travel time errors into subsequent steps. Once satisfactory gathers are generated, manual direct arrivals picking can be performed. In our case, as we had good enough source depths from the drilling data and due to the high computational cost of running backpropagation, it was only run for a few depths for quality control. However, in the absence of source depths, the only way to obtain them is through backpropagation.

## 6. SWD Applications

The specialized processing workflow enabled the successful conversion of passive seismic while drilling data into active equivalents, which in turn enables various applications such as travel time tomography and imaging.

### 6.1. Travel Time Tomography

**Figure 14:**
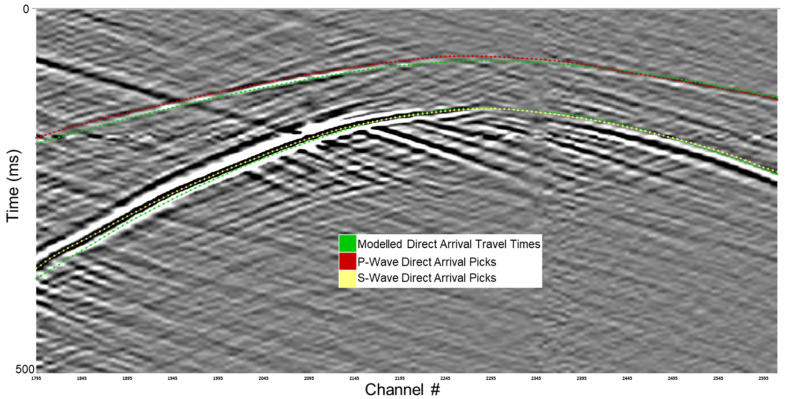
Guided first break picking of the P-wave and S-wave direct arrivals. The modelled P-wave and S-wave direct arrival times appear in green, while the picked travel times appear in red and yellow, respectively.

The calculated P-wave and S-wave direct arrival travel times guide the first break picking of the 10 m binned data, as shown for a shallow depth source location in Figure 14. The P-wave and S-wave direct arrivals stand out easily and are easy to pick, particularly in the shallower source gathers. The actual picked travel times were close to those modelled using the initial velocity model.

**Figure 15:**
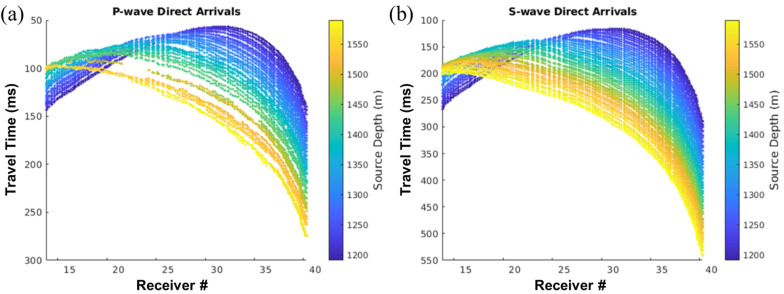
Travel time picks of (**a**) P-wave and (**b**) S-wave direct arrivals for all source depths.

However, P-wave arrivals became more challenging to recognize and pick for deeper depths, resulting in fewer picks than S-waves, as shown in Figure 15, where only the picks every 10 m in receiver depth were displayed, as the inversion is set to a 10 m spacing in the offset direction.

**Figure 16:**
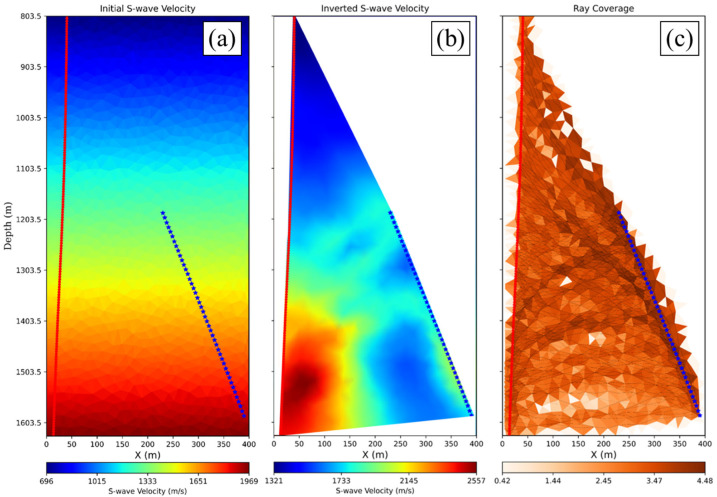
The application of shear wave travel time tomography using (**a**) an initial linear gradient model, showing (**b**) the resulting inverted cross-well S-wave velocity profile, and (**c**) a normalized ray coverage map. The red dots and blue stars correspond to the locations of receivers and sources, consecutively.

The travel time picks and source-receiver geometries were prepared for running the tomography using the open-source pyGimli package (version 1.5.4) [49]. Because we did not pick the P-wave direct arrivals, especially for deeper sources, we expect high uncertainties. The initial S-wave velocity model was created using a linear gradient with increasing velocities with depth, as shown in Figure 16a, with the receiver array geometry overlaid in red stars at 5 m depth spacing and the source geometry in blue stars at 10 m measured depth spacing. We ran the travel time tomography inversions, which converged quickly. The initial result of the inverted S-wave velocity cross-well profile is shown in Figure 16b, while Figure 16c shows a normalized ray coverage map.

**Figure 17:**
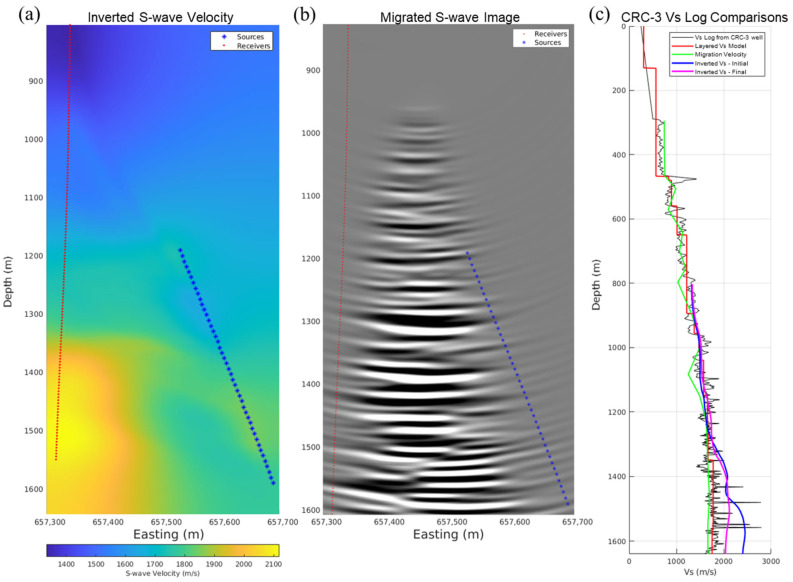
The final (**a**) updated inverted S-wave velocity profile limited to upgoing rays, and (**b**) Kirchhoff migrated the S-wave cross-well image using the inverted S-wave velocity profile, and (**c**) validation of inverted S-wave velocity logs at the CRC-3 well location, demonstrating better agreement with the original log.

Due to the lower data quality for receivers below the depth level of the source, resulting in higher uncertainties in the inversion, we dropped the corresponding S-wave direct arrivals travel time picks, and reran the inversion. The inverted model in Figure 17a showed a closer fit to the general trend of the CRC-3 S-wave velocity log, plotted in magenta, than the initial inverted profile, plotted in blue, as shown in Figure 17c. These preliminary tomography results have plenty of room for improvement, incorporating better quality checks in subsequent studies.

### 6.2. SWD Imaging

We then used the resulting S-wave velocity profile to migrate the upgoing S-wave reflections after muting the direct arrivals, using 2D cross-well Kirchhoff migration. The migrated S-wave image in Figure 17b shows the achieved cross-well image profile. The implemented imaging workflow can be run efficiently while drilling, without requiring enormous computing power to provide real-time cross-well images and ahead-of-the-bit predictions.

## 7. Discussion

Distributed Acoustic Sensing (DAS) is an enabling technology in this experiment, enabling the continuous recording of passive seismic data. The use of an engineered fiber at a distributed seismic sensor also enabled the acquisition of high-quality seismic data at high spatial and temporal sampling. Installing fiber-optic cables permanently in a borehole comes with a huge advantage compared to the use of conventional downhole geophones, as fiber-optic cables can cover the entire well length without the need to redo the acquisition at different levels. This is particularly important for passive data acquisition, as the target signals are not controlled and do not repeat. However, DAS data acquisition generates huge datasets during continuous recording, which pushes the limits of existing hardware and software, requiring special attention to handle. On the other hand, fiber-optic cables have an inherent limitation due to their directional sensitivity, as they are most sensitive to motion along their length. This limitation manifests itself in the form of dim measurements arriving at a normal incident to the fiber-optic cable. This limitation can reduce the pickable parts of the wavefield in one gather for tomography applications; however, the huge amount of data compensates for that. This section discusses the conducted research and outlines key factors considered to decipher the acquired DAS dataset and associated uncertainties.

### 7.1. Pilot Signal Recording

The acquired passive seismic dataset had no pilot signal recording, complicating its analysis. Seismic data recorded during drilling result from the interaction between the drill bit and the drilled rocks. Every time the drill bit rotates and scrapes off a bit of the drilled rock, it generates acoustic energy. The strength of the acoustic signals varies depending on the applied weight on bit, and their frequency varies depending on the drill bit rotation rate (RPM) and possibly many other factors such as rock properties. Two approaches enable the understanding and analysis of the acquired SWD data: modelling or processing. The modelling approach requires recording good-quality and carefully synchronized rig drilling data. It also requires prior knowledge of the hardness of the formation rocks being drilled. Preferably, processing requires recording or estimating the drill bit’s signature as close as possible to the bit-rock interface. The latter approach is more straightforward, where rig drilling data can also be helpful in quality-checking the processing.

### 7.2. Passive Source Location

Accurate knowledge of the passive source, drill bit, and location in depth is key to associating recorded passive data at each depth. Drilling data provides a reasonably accurate depth estimation of the drill bit, based on the number of drilling rods used, which can be easily converted to vertical depth given a known drilling trajectory. A backpropagation approach can be used to improve or even estimate the drill bit depth without drilling data. The availability of acceptable rig drilling data enabled the described processing workflow. We used sufficiently accurate source depth estimates to transform passive seismic data into an active equivalent, which we then utilized for backpropagation to enhance the source depth estimate further. The backpropagation can be based on P-wave, S-wave velocities, direct arrivals, or both. P-wave-based backpropagation provided accurate source location estimates, particularly in depth. S-wave-based backpropagation provided improved source depth estimates, particularly offset from the observation well. Ultimately, combining both yields better source location estimates in terms of depth and offset. As the processing workflow depends only on source depth, and for the sake of faster computations, we limited the focus to source depth estimation.

### 7.3. Informed Data Processing

Prior knowledge of subsurface velocity variations with depth aids in computing travel times, which we then used to guide the processing of the SWD data and the first break picking of the processed data. This approach is an alternative to recording the pilot signal of the passive source. This alternative approach is associated with some uncertainties.

### 7.4. Associated Uncertainties

As with every SWD processing step, there is some degree of uncertainty, which can be quantified and minimized through iterative refinement. The uncertainty starts much earlier during the data acquisition and subsequently in processing algorithms used to process the data. For example, the geometry projection step, used to simplify subsequent processing steps, introduces minor errors in travel times. The 2D plane used to project the actual geometry to is chosen carefully to minimize these errors. The estimation of the drill bit depth, which we used in estimating a pilot trace representing the drill bit signature, we then removed from the data by deconvolution. Deconvolution with the estimated pilot considers the relative kinematics but does not account for the correct travel times to all receivers from a source in depth. The initial S-wave velocity model, based on which we computed travel times, then used to apply time delay corrections after deconvolution. Given the assumption that the drill bit does not drill too fast in a short period, we performed depth binning to help improve data quality by stacking. We then used the stacked data to estimate more accurate drill bit depths and update the initial velocity through backpropagation. The velocity update helps prevent the propagation of errors further due to the use of a potentially inaccurate initial velocity profile. As drill bit depths increase monotonically, the progression of estimated source depths can be used as a quality control step to ensure consistency. The updated depths and velocities can then be used to rerun the deconvolution and obtain new time delay corrections iteratively until satisfactory gathers are obtained.

However, as the source energy is variable and uncontrolled, depth bin stacking accumulates different source energy levels for each depth interval, as some depth levels would take more time than others to be drilled. The variable source energy is not an issue here, as preserving the relative energy of the source at each depth is not required. However, source energy equalization can be used to ensure the stability of subsequent processing steps. All the factors discussed above are variables that contribute to the associated uncertainties.

## 8. Conclusions

We developed a specialized workflow to process passive seismic data acquired using downhole DAS in a monitoring well while drilling a second well. The developed workflow combined several data sources, including continuous seismic data, drilling rig sensing, deviation surveys, and initial subsurface property models.

We used initial subsurface models, including P-wave and S-wave velocities and density, to perform travel time modelling, guiding the processing workflows and elastic seismic modelling for further validation. We also applied recent advances in machine learning-based denoising methods to remove most of the DAS instrument noise before applying standard denoising algorithms to remove other types of noise, such as spikes, horizontal striping, and very low and high-frequency noises. As the drill bit source signature (pilot trace) recordings are not available, processing the passive seismic data is more challenging. Converting them into active equivalents makes them easier to understand and interpret. Given recorded pilot signals, the conversion is typically achieved through cross-correlation, deconvolution, and the application of a time correction to transform the continuously recorded passive data into active seismic equivalents with correct source onset times. Hence, we used a data-driven approach to estimate a pilot trace for cross-correlation and deconvolution. Alternatively, we derived time corrections using travel time modelling based on initial knowledge of subsurface velocities and an estimated source depth from the rig data. At this stage, we converted each continuous record into active equivalents, but with a low and variable signal-to-noise ratio and uneven source depth spacing, primarily due to the variable nature of the drill bit’s interactions with the drilled rocks and the variable penetration rates. Binning these sources into regular depth bins results in improved and consistent SNRs and provides better data quality for further processing. Only at limited source depths, we further enhanced the initial time corrections by backpropagation to improve the source depth estimation and the associated time corrections.

Subsequently, we manually picked the P-wave and S-wave first arrivals and used them to perform travel time tomography to subsequently reconstruct the P-wave and S-wave cross-well velocity profiles. We then used these velocity profiles for the imaging of Vertical Seismic Profiling (VSP) data. We separated different waveform components before performing cross-well Kirchhoff migration.

The implementation of specialized processing workflows was successful mainly due to careful integration of several complementary data sources, travel time modelling and reverse time propagation to estimate source depths and travel time corrections accurately. The resulting deconvolved source gathers were used to invert for subsurface cross-well velocity profiles and VSP imaging. The conversion of the passive SWD data into active equivalents enables the application of the deep-learning approach described in the next chapter. These promising results pave the way for more extensive studies and potential applications to field data, where the goal is to simplify data acquisition and save costs. Specifically, this proof-of-concept work enables various drilling and mining monitoring operations in an SWD context, including prediction ahead of the bit, real-time imaging, and updating subsurface models, thereby providing informed decisions on Drill-Hole Locations and Trajectories.

## Figures and Tables

**Table 1:** Data acquisition timeline of the SWD DAS data.

Date	Number of Records	Channels/Record	Activity	CRC-4 Section	Monitor Well
22 July 2019	2880	5248	Drilling	Shallow	CRC-2
23 July 2019	2880	5248	Drilling	Shallow	CRC-2
24 July 2019	2880	5248	Drilling	Shallow	CRC-2
25 July 2019	2880	5248	Drilling	Shallow	CRC-2
26 July 2019	245	5248	Non-Drilling	Shallow	CRC-2
27 July 2019	2726	1408	Non-Drilling	Deep	CRC-3
28 July 2019	2864	1408	Non-Drilling	Deep	CRC-3
28 July 2019	54	2816	Drilling	Deep	CRC-3
29 July 2019 ^1^	2880	2816	Drilling	Deep	CRC-3
30 July 2019	2880	2816	Non-Drilling	Deep	CRC-3
31 July 2019	2879	2816	Non-Drilling	Deep	CRC-3

^1^ Subset of the dataset used for demonstration.

## Data Availability

Restrictions apply to the availability of these data. Data were obtained from the CO2CRC project and are available to its members. The membership can be requested at info@co2crc.com.au.

## Abbreviations

The following abbreviations are used in this manuscript:

SWD	Seismic While Drilling
DAS	Distributed Acoustic Sensing
BHA	Bottom Hole Assembly
VSP	Vertical Seismic Profiling
RVSP	Reverse Vertical Seismic Profiling
2D	Two-Dimensional
PDC	Polycrystalline Diamond Compact
RMS	Root Mean Square
MD	Measured Depth
ROP	Rate of Penetration
WOB	Weight on Bit
RPM	Rotations Per Minute

## References

[1] Meehan R., Miller D., Haldorsen J., Kamata M., Underhill B. (1993). Rekindling Interest in Seismic While Drilling. Oilfield Rev..

[2] Miranda F., Aleotti L., Abramo F., Poletto F., Craglietto A., Persoglia S., Rocca F. (1996). Impact of the Seismic “While Drilling” Technique on Exploration Wells. First Break.

[3] Naville C., Serbutoviez S., Throo A., Vincké O., Cecconi F. (2004). Seismic While Drilling (Swd) Techniques with Downhole Measurements, Introduced by Ifp and Its Partners in 1990–2000. Oil Gas Sci. Technol. Rev. IFP Energ. Nouv..

[4] Anchliya A. A Review of Seismic While Drilling (SWD) Techniques: A Journey from 1986 to 2005. Proceedings of the SPE Europec/EAGE Annual Conference and Exhibition.

[5] Zhou B., Mason I., Greenhalgh S., Subramaniyan S. (2015). Seeing Coal-Seam Top Ahead of the Drill Bit through Seismic-While-Drilling. Geophys. Prospect..

[6] Tekinalp O., Ulsoy A.G. (1989). Modeling and Finite Element Analysis of Drill Bit Vibrations. J. Vib. Acoust..

[7] Ma D., Zhou D., Deng R. The Computer Simulation of the Interaction Between Roller Bit and Rock. Proceedings of the International Meeting on Petroleum Engineering.

[8] Spanos P.D., Sengupta A.K., Cunningham R.A., Paslay P.R. (1995). Modeling of Roller Cone Bit Lift-Off Dynamics in Rotary Drilling. J. Energy Resour. Technol..

[9] Rector J.W., Marion B.P., Widrow B. Use of Drill-Bit Energy As a Downhole Seismic Source. Proceedings of the SEG Technical Program Expanded Abstracts.

[10] Rector J.W., Marion B.P., Hardage B.A. (1989). The Use of an Active Drill Bit for Inverse VSP Measurements. Explor. Geophys..

[11] Rector J.W. (1990). Utilization of Drill-Bit Vibrations as A Downhole Seismic Source. Ph.D. Thesis.

[12] Sui D. (2025). Real-Time Drilling Performance Optimization Using Automated Penetration Rate Algorithms with Vibration Control. Fuels.

[13] Sudev L.J., Ravindra H.V. (2009). Tool Wear Estimation in Drilling Using Acoustic Emission Signal by Multiple Regression and GMDH. ASME 2008 International Mechanical Engineering Congress and Exposition, Boston, MA, USA, 31 October–6 November 2008.

[14] Auriol J., Kazemi N., Niculescu S.-I. (2021). Sensing and Computational Frameworks for Improving Drill-String Dynamics Estimation. Mech. Syst. Signal Process..

[15] Rector J.W., Hardage B.A. (1992). Radiation Pattern and Seismic Waves Generated by a Working Roller-Cone Drill Bit. Geophysics.

[16] Asanuma H., Niitsuma H. Triaxial Inverse VSP Uses Drill Bits As a Downhole Seismic Source. Proceedings of the SEG Technical Program Expanded Abstracts.

[17] Haldorsen J.B.U., Miller D.E., Walsh J.J. (1995). Walk-Away VSP Using Drill Noise as a Source. Geophysics.

[18] Aleotti L. (1999). Seismic While-Drilling Technology: Use and Analysis of the Drill-Bit Seismic Source in a Cross-Hole Survey. Geophys. Prospect..

[19] Poletto F. (2005). Energy Balance of a Drill-Bit Seismic Source, Part 1: Rotary Energy and Radiation Properties. Geophysics.

[20] Poletto F. (2005). Energy Balance of a Drill-Bit Seismic Source, Part 2: Drill-Bit versus Conventional Seismic Sources. Geophysics.

[21] Poletto F., Miranda F., Corubolo P., Schleifer A. Drill Bit as a Seismic Source for Near-well Imaging. Proceedings of the SEG Technical Program Expanded Abstracts 2008.

[22] Hardage B.A. (2009). Seismic-While-Drilling: Techniques Using the Drill Bit as the Seismic Source.

[23] Egorov A., Bakulin A., Silvestrov I., Golikov P. (2021). Research Note: Automated Data-Driven Alignment of near-Bit and Top-Drive Vibration Sensors for Seismic While Drilling and Beyond. Geophys. Prospect..

[24] Silvestrov I., Bakulin A., Aldawood A., Hemyari E., Egorov A. (2022). Improving Shallow and Deep Seismic-While-Drilling with a Downhole Pilot in a Desert Environment. Geophysics.

[25] Deily F.H., Dareing D.W., Paff G.H., Ortloff J.E., Lynn R.D. (1968). Downhole Measurements of Drill String Forces and Motions. J. Eng. Ind..

[26] Egorov A., Golikov P., Silvestrov I., Bakulin A. (2020). Alignment and Comparison of Top Drive and Near-Bit Accelerometers for Seismic-While-Drilling. Saint Petersburg 2020.

[27] Rector J.W., Marion B.P. (1991). The Use of Drill-bit Energy as a Downhole Seismic Source. Geophysics.

[28] Yoon B., Park K.G., Lee C., Yang K., Song Y., Lee T.J., Park I. Pre-Processing for Seismic While Drilling Data with Unfavorable Pilot Condition Acquired in the Vicinity of Geothermal Well in Pohang, Korea. Proceedings of the World Geothermal Congress 2015.

[29] Silvestrov I., Hemyari E., Andrey B., Luo Y., Aldawood A., Poletto F., Liu Y., Du Y., Egorov A., Golikov P. Processing of Seismic-While-Drilling Data from the DrillCAM System Acquired with Wireless Geophones, Top-Drive, and Downhole Vibrations Sensors. Proceedings of the SPE Middle East Oil & Gas Show and Conference.

[30] Wang L., Liu H., Tong S., Yin Y., Xing L., Zou Z., Xu X. (2015). Retrieving Drill Bit Seismic Signals Using Surface Seismometers. J. Earth Sci..

[31] Poletto F., Miranda F., Corubolo P., Schleifer A., Comelli P. (2014). Drill-bit Seismic Monitoring While Drilling by Downhole Wired-pipe Telemetry. Geophys. Prospect..

[32] Asgharzadeh M., Urosevic M., Bona A., Grant A., Poletto F. (2019). Drill Bit Noise Imaging without Pilot Trace, a near Surface Interferometry Example. Solid Earth.

[33] Ivandic M., Kaslilar A., Juhlin C. (2022). Subsurface Seismic Imaging with a Hammer Drilling Source at an Exploration Drilling Test Center in Örebro, Sweden. Adv. Geosci..

[34] Bellezza C., Goertz A., Bergfjord E.V., Lindgård J.E., Corubolo P., Poletto F., Moskvil L.M. (2021). Seismic-While-Drilling by Drill-Bit Source and Large-Aperture Ocean-Bottom Array. Geophysics.

[35] Liu Y., Draganov D., Wapenaar K., Arntsen B. (2016). Retrieving Virtual Reflection Responses at Drill-Bit Positions Using Seismic Interferometry with Drill-Bit Noise. Geophys. Prospect..

[36] Xu L., Chen H., Zhang X., Wang X. (2018). Green’s Function Retrieval with Marchenko and Inter-Source Seismic Interferometry Method for Drill-Bit Seismic While Drilling. J. Geophys. Eng..

[37] Landa E., Sinitsin P., Shustak M., Reshef M. (2018). Time-Reversal Scatterer Detection in 3D Media Using Kirchhoff Back Propagation. SEG Technical Program Expanded Abstracts 2018, Proceedings of the 2018 SEG International Exposition and Annual Meeting, Anaheim, CA, USA, 14–19 October 2018.

[38] Shustak M., Landa E. (2017). Time Reversal Based Detection of Subsurface Scatterers. SEG Technical Program Expanded Abstracts 2017.

[39] Zhebel O., Gajewski D., Vanelle C. (2011). Localization of Seismic Events in 3D Media by Diffraction Stacking. SEG Technical Program Expanded Abstracts 2010, Proceedings of the SEG 2010: Society of Exploration Geophysicists Annual Meeting, Denver, CO, USA, 17–22 October 2010.

[40] Butt C.R.M. (1998). Supergene Gold Deposits in the Yilgarn Craton. AGSO J. Aust. Geol. Geophys..

[41] Kerr T.L., O’Sullivan A.P., Podmore D.C., Turner R., Waters P. (1994). IRON: Geophysics and Iron Ore Exploration: Examples from the Jimblebar and Shay Gap-Yarrie Regions, Western Australia. ASEG Ext. Abstr..

[42] Pevzner R., Tertyshnikov K., Sidenko E., Ricard L. (2020). Monitoring Drilling and Completion Operations Using Distributed Acoustic Sensing: CO2CRC Stage 3 Project Case Study. First EAGE Workshop on Fibre Optic Sensing.

[43] Kroon D.-J. Accurate Fast Marching. https://au.mathworks.com/matlabcentral/fileexchange/24531-accurate-fast-marching.

[44] Fink M. (1992). Time Reversal of Ultrasonic Fields. I. Basic Principles. IEEE Trans. Ultrason. Ferroelectr. Freq. Control..

[45] Collet O., Gu X., Tertyshnikov K., Pevzner R. (2024). Removing DAS Hardware Noise: A Comparison Between Traditional and Weakly Supervised Machine Learning Approaches. 85th EAGE Annual Conference & Exhibition.

[46] Qin Z., Urosevic M., Pevzner R. (2020). Distributed Acoustic Sensing Technique for Seismic While Drilling: Stage 3 of the CO2CRC Otway Project Case Study. EAGE Workshop on Fiber Optic Sensing for Energy Applications in Asia Pacific.

[47] Qin Z., Urosevic M., Bona A., Pevzner R., Tertyshnikov K. A New Method for Determining Drill-Bit Signal Emission Time. Proceedings of the 2021 Australasian Exploration Geoscience Conference (AEGC 2021).

[48] Al-Hemyari E., Collet O., Tertyshnikov K., Pevzner R. (2024). Supervised Deep Learning for Detecting and Locating Passive Seismic Events Recorded with DAS: A Case Study. Sensors.

[49] Rücker C., Günther T., Wagner F.M. (2017). pyGIMLi: An Open-Source Library for Modelling and Inversion in Geophysics. Comput. Geosci..

